# EF4 knockout *E. coli* cells exhibit lower levels of cellular biosynthesis under acidic stress

**DOI:** 10.1007/s13238-014-0050-3

**Published:** 2014-04-05

**Authors:** Fang Yang, Zhikai Li, Jia Hao, Yan Qin

**Affiliations:** 1 Laboratory of Noncoding RNA, Institute of Biophysics, Chinese Academy of Sciences, Beijing, 100101 China; 2 University of Chinese Academy of Sciences, Beijing, 100049 China

**Dear Editor,**

Recently, a novel elongation factor, termed EF4, was identified in *E. coli* and shown to catalyze the back translocation of the E- and P-tRNAs (tRNA at E and P sites) to A- and P-sites (Qin et al., 2006). Despite a great number of recent biochemical studies, little remains known as to the precise physiological role of EF4 *in vivo*. In *E. coli*, 80% of EF4 molecules are associated with the cell membrane under optimal conditions, but when cells grow at pH 6 in media containing 100 mmol/L MgCl_2_, this percentage is reduced to less than 20% (Pech et al., 2011). Furthermore, it was revealed that overexpression of EF4 in *E. coli* severely affected cell growth (Qin et al., 2006). However, EF4 knockout (∆EF4, KO) cells exhibited no apparent phenotype in rich LB medium (Shoji et al., 2010). Under certain stresses, such as low temperature or high ionic stress, ∆EF4 cells were out-competed by wildtype (WT) cells within a short period of time (Shoji et al., 2010; Pech et al., 2011).

The only organism for which EF4 was shown to be essential is the gastritis pathogen *Helicobacter pylori*. A systematic knockout analysis in *H. pylori* revealed that EF4 was essential for cell survival at hostile pH levels close to the acidic conditions in the stomach (Bijlsma, 2000). We therefore wondered if low pH values represent a stress condition under which EF4 is required in *E. coli*. Because the *ef4* KO strain in Keio was not designed from the wildtype BW25113 (Karim, 2010), we deleted *ef4* in BW25113 strain by ourselves using the λ Red system (Datsenko and Wanner, 2000) (Fig. 1A).

**Figure 1:**
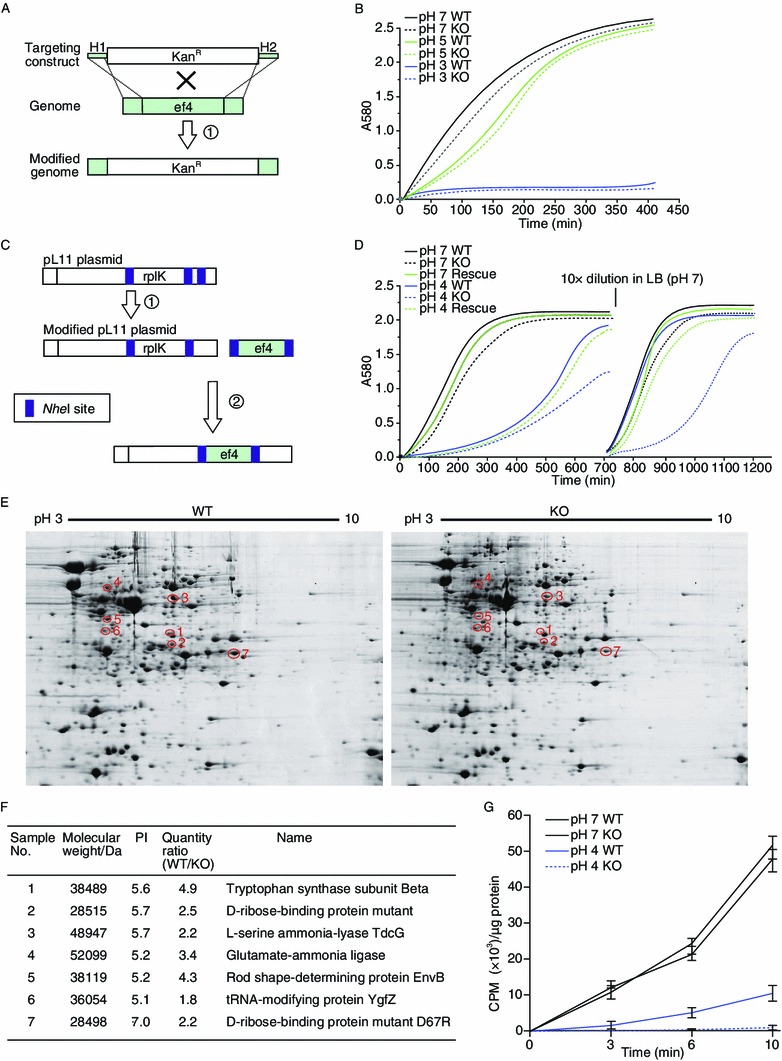
**Effects of low pH on the growth and protein translation of*ef4*-deleted cells**. (A) Construction of a single-gene deletion mutant using the λ Red system. H1 and H2: homologous to chromosomal sequences of the deleted gene; Step ① indicates that the λ-mediated recombination. The targeting construct containing selection marker Kar^R^ was transferred to strain BW25113, which contained plasmids expressing the components of λ Red system. Then homologous recombination was performed, and the genomic *ef4* gene was replaced by Kar^R^. Finally, the resistance gene was eliminated using a helper plasmid. (B) *E. coli* WT cells and ΔEF4 cells (KO) were grown in LB medium at pH 3, 5 or 7. (C) Construction of the EF4 rescues plasmid. Step ① indicates the redundant *Nhe*I restriction site of pL11 plasmid containing tac promoter was mutated by PCR amplification. Step ② indicates the *ef4* gene amplified from *E. coli* genomic DNA and mutant pL11 digested by *Nhe*I enzyme before ligation to form the EF4 rescue plasmid. (D) *E. coli* WT cells, ΔEF4 cells, and rescued cells were grown in LB medium at either pH 4 or pH 7. After cells reached stationary phase, each of the samples was diluted 10 times in LB medium (pH 7). (E) 2-DE images of total proteins in *E. coli* at pH 4. The number in red circle represents the number of proteins downregulated in ΔEF4 cells. (F) Quantitative analysis of differentially expressed protein spots from (E). (G) Analysis of translation efficiency *in vivo* by 35S-methionine incorporation. At times indicated, samples were taken from each culture, and the incorporation of radioactive material was determined

In order to establish a relationship between EF4 dependency and pH conditions, we first prepared LB medium with a pH of 3, 5, or 7 and observed the growth of WT or ΔEF4 (KO) cells at each pH. As shown in Fig. 1B, KO cells grew as well as WT cells at both pH 7 and pH 5, but neither could survive at pH 3. We then found a difference in cell growth at pH 4 (Fig. 1D). WT cells grew vigorously after a relatively long lag phase, and finally reached saturation at an OD of 2 (A_580_). In contrast, KO cells remained in lag phase for an extended period of time, to finally reach saturation at about OD_580_ = 1. After a 10-fold dilution with pH 7 medium, WT cells regained their normal growth, comparable to that observed for cells grown continuously at pH 7. In contrast, the KO strain underwent a relatively long lag phase even after increase of pH, and reached saturation at about OD_580_ = 2, as observed for WT cells (Fig. 1D). We further found ΔEF4 cells with a complementary plasmid survived under pH 4 and regain similar growth curves to WT cells under the same conditions (Fig. 1C and 1D). These results clearly demonstrate that EF4 is required for the *E. coli* cell growth at pH 4.

To investigate the overall impact of EF4 deletion on the protein profile of WT or ΔEF4 cells, two-dimensional electrophoresis (2-DE) was employed. No protein changes were detected at pH 7 (Fig. S1). Intriguingly, despite the significant slow-down in growth, only six proteins were downregulated in KO cells at pH 4 (Fig. 1E and 1F). Spots 1–7 were identified as tryptophan synthase subunit β (TrpB), D-ribose-binding protein mutant, glutamate-ammonia ligase, L-serine ammonia-lyase (TdcG), rod shape-determining protein (MreB), tRNA-modifying protein (YgfZ), and D-ribose-binding protein mutant D67R, respectively. TrpB catalyzes the last step of the tryptophan biosynthesis (Lane and Kirschner, 1991). D-ribose-binding protein serves as the primary chemoreceptor for chemotaxis (Bjorkman et al., 1994). Glutamate-ammonia ligase catalyzes the single reaction in the glutamine biosynthesis pathway (Keseler et al., 2013). Three genes (*sdaA*, *sdaB*, *tdcG*) code for three l-serine deaminases in *E. coli*, and participate in glycine, serine, and threonine metabolism and cysteine metabolism, and null mutant cells for all three genes grow normally in glucose minimal medium. However, the mutant cells become very large, and many lyse upon subculturing in the same medium lacking tryptophan (Zhang and Newman, 2008). Deletion of *mreB* disrupts the typical shape of *E. coli*, enlarging them before finally lysing these cells (Kruse et al., 2005). YgfZ protein modifies tRNAs, and its deletion mutant grows slowly relative to WT, especially at low temperatures (Ote et al., 2006). In summary, these proteins are involved in protein biosynthesis, gluconeogenesis, and cell metabolism processes. Intriguingly, however, none of the six proteins are essential for *E. coli* growth. We speculate that KO cells do not display signs of physical damage, and can be rescued from growth arrest upon subculturing in medium of pH 7. Hence, when grown to the same A_580_, the cells showed similar protein expression profiles.

Other conditions that lead to a slowing in growth upon *ef4* KO were shown to result in decreased translation (Pech et al., 2011). Similar phenotypes were observed in yeast, where EF4 was found to be located inside the mitochondria (Bauerschmitt et al., 2008). In yeast, loss of EF4 function caused growth defects under conditions of low temperature and starvation, and importantly, decreased mitochondrial translation (Bauerschmitt et al., 2008). In order to investigate whether translation was decreased in our EF4 KO strain at pH 4 we measured the S^35^-methionine incorporation rate to assay protein translation *in vivo*. Our analysis revealed that protein synthesis was inhibited at pH 4 even in WT cells, with levels of protein synthesis at about 20% relative to that at pH 7 (Fig. 1G). This result was consistent with the slower growth found in WT cells at pH 4. Under normal pH 7 conditions, loss of EF4 made no significant difference (Fig. 1G). Taken together, these findings suggested a translation defect present in the ΔEF4 strain at pH 4. As a consequence, fewer amid acids and tRNAs are needed, which in turn induces a decrease in expression of proteins participating in these biosynthesis processes, such as TrpB, glutamate-ammonia ligase, TdcG, and YgfZ. This decrease in translation further impairs the response of *E. coli* cells to the acidic growth conditions, and the expression of proteins required directly or indirectly for adaption to this environment also changed (Fig. 1E and 1F). Collectively, our results suggested that the lack of EF4 affects translation and growth only in unfavorable conditions. Importantly, its high degree of sequence conservation throughout evolution indicates that EF4 may play essential roles for the survival in unfavorable growth conditions other than extreme temperature, high metal concentrations, or low pH, and be in part an evolutionary response to temporary/transient unfavorable conditions.

Because of the association of EF4 with ribosomal movement, and the roles of EF4 in translational processes, we examined the polysome patterns of ΔEF4 cells throughout the complete growth process, including lag phase (A_580_ = 0.1), log phase (A_580_ = 0.4–0.6), semi-log phase (A_580_ = 0.8), and stationary phase (A_580_ = 1.0). When cells grew at pH 7, the polysome profile of ΔEF4 cells was similar to that of WT cells (Qin et al., 2006). Cells in the log phase (A_580_ = 0.4–0.6) had an active translation system and protein biosynthesis was efficient. All the classical peaks of ribosomes or ribosomal subunits were present under normal conditions pH 7 (Fig. 2A and 2C). When the pH was lowered to 4, WT cells showed the similar polysome pattern to that at pH 7 (Fig. 2A and 2B). However, the quantity of ribosomes in ΔEF4 cells was reduced to 40% of that at A_580_ = 0.1, and some non-classical ribosomal subunits appeared as indicated with arrows in the top panel of Fig. 2D. Compared to the position of the 30S peak in Fig. 2C, the non-classical peak had a smaller sedimentation coefficient, indicating that the biogenesis of the 30S subunit was hindered. This situation became more profound when A_580_ reached 0.4. In addition to premature 30S subunits, there were premature 50S peaks, as shown in panel 2 of Fig. 2D. As A_580_ reached 0.6, there were no further non-classical peaks, but the total quantity of ribosomes did not increase relative to that at A_580_ = 0.1. In addition, the ratio between polysome and 70S ribosomes suggests that the translation efficiency was low. At A_580_ ≥ 0.8, ΔEF4 cells had the same polysome pattern, but exhibited lower levels of polysome and 70S ribosomes than that at pH 7, and the total quantity of ribosomes was much lower than the 4S fraction (Fig. 2D). We also noted that the total quantity of ribosomes in ΔEF4 cells from different growth phases was always much lower than that of 4S, which represents the background absorbance of cell lysates at 260 nm (Fig. 2). Our results suggested that ΔEF4 cells took 10 h to overcome subunit maturation defects, reflecting the inhibition of ribosomal biogenesis in ΔEF4 cells at pH 4. In contrast, the WT strain succeeded in overcoming low pH-induced problems, even though the lag phase was prolonged to 6 h (Figs. 1 and 2). This data also provides a possible explanation for the null-phenotype observed by Shoji et al. (Shoji et al., 2010), who analyzed pH conditions ranging from 3.5 to 12. The difference observed here between the two strains only became apparent after 7 h of culture. In addition, the 70S ribosomes and the 30S/50S subunits had reached an equilibrium indicating that the translation machinery will not work efficiently (Nierhaus and Wilson, 2004). In addition, rescued ΔEF4 cells showed classical polysome patterns (Fig. 2E and 2F).

**Figure 2:**
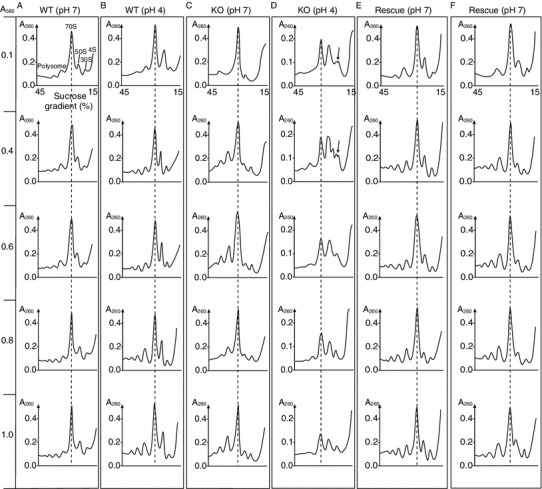
**Polysome profile of ΔEF4*E. coli*cells during different growth phases at pH 7 and pH 4**. Cell extracts were prepared from ΔEF4 strains during different growth phases (OD_580_ = 0.1, 0.4, 0.6, 0.8 or 1.0). The gradient was fractionated while monitoring the absorbance at 260 nm (A_260_). The dotted vertical line indicates the monomer peak. (A and B) Polysome profiles of WT cells grown at either pH 7 or pH 4. The ribosome peak-subunits (30S, 50S), monomer (70S) and polysome, and 4S peak are indicated. The polysome pattern of cells growing at pH 4 showed no obvious difference to those from cells growing under normal conditions. (C) Polysome profiles of ΔEF4 *E. coli* cells grown at pH 7. (D) Polysome profile of ΔEF4 *E. coli* cells grown at pH 4. Black arrows indicate non-classical ribosomal subunits. Note that units on the Y-axis for pH 4 samples are half of those for the pH 7 sample. The quantity of ribosomes in pH 4 samples was significantly reduced compared to that in pH 7 with only few polysomes detected. (E) Polysome profile of KO cells overexpressing EF4 grown at pH 7. The ribosome peaks showed nearly the same classical patterns as WT at pH 7. (F) Polysome profile of EF4-rescued KO cells grown at pH 4. The polysome quantity and patterns under this condition did not change significantly compared to those of cells grown at pH 7. However, the polysome quantity was higher than that in ΔEF4 *E. coli* cells at pH 4, with polysome patterns more classical

In summary, our results showed slower growth of ΔEF4 cells and a lower level of intracellular biosynthesis under acidic stress. In agreement with growth defects, the EF4-depleted strain exhibited a very slower protein translation at pH 4 *in vivo*. However, there was no difference between WT and ΔEF4 cells at normal condition pH 7 (Figs. 1 and 2). This phenotype suggested that EF4 plays an essential and specific role in increasing the fitness of *E. coli* under unfavorable to extreme environmental conditions, where it ensures appropriate/adequate regulation of protein synthesis. Our data presented here provide the first time evidence that the translation factor EF4, contribute to protein synthesis and cell growth at unfavorable conditions, explaining why no apparent role was described at optimal growth conditions before.

## Electronic supplementary material

Below is the link to the electronic supplementary material.

Supplementary material 1 (PDF 510 kb)
