# Supplementary material for: EF4 knockout E. coli cells exhibit lower levels of cellular biosynthesis under acidic stress
Source: Protein Cell. 2014 Apr 5;5(7):563–7. doi: 10.1007/s13238-014-0050-3 (PMC4085283; doi:10.1007/s13238-014-0050-3)
Supplement: Supplementary file 1 — Supplementary material 1 (PDF 510 kb) [file 13238_2014_50_MOESM1_ESM.pdf]

SUPPLEMENTARY MATERIALS

EF4 knockout *E. coli* cells exhibit lower levels of cellular biosynthesis under acidic stress

Fang Yang[1,2], Zhikai Li[1], Jia Hao[1], and Yan Qin[1,*]

[1] Laboratory of Noncoding RNA, Institute of Biophysics, Chinese Academy of Sciences, 15 Datun Road, Chaoyang District, Beijing 100101, China

[2] University of Chinese Academy of Sciences, Beijing 100049, China

Corresponding author: Yan Qin, at the Laboratory of Noncoding RNA, Institute of Biophysics, Chinese Academy of Science, Beijing 100101, China; Tel: 010-64869250; Fax: 010-64869250; E-mail: *qiny@ibp.ac.cn*

Running title: EF4 is essential for optimal growth under low pH stress

KEYWORDS: ribosome, translation regulation, elongation factor 4 (EF4)

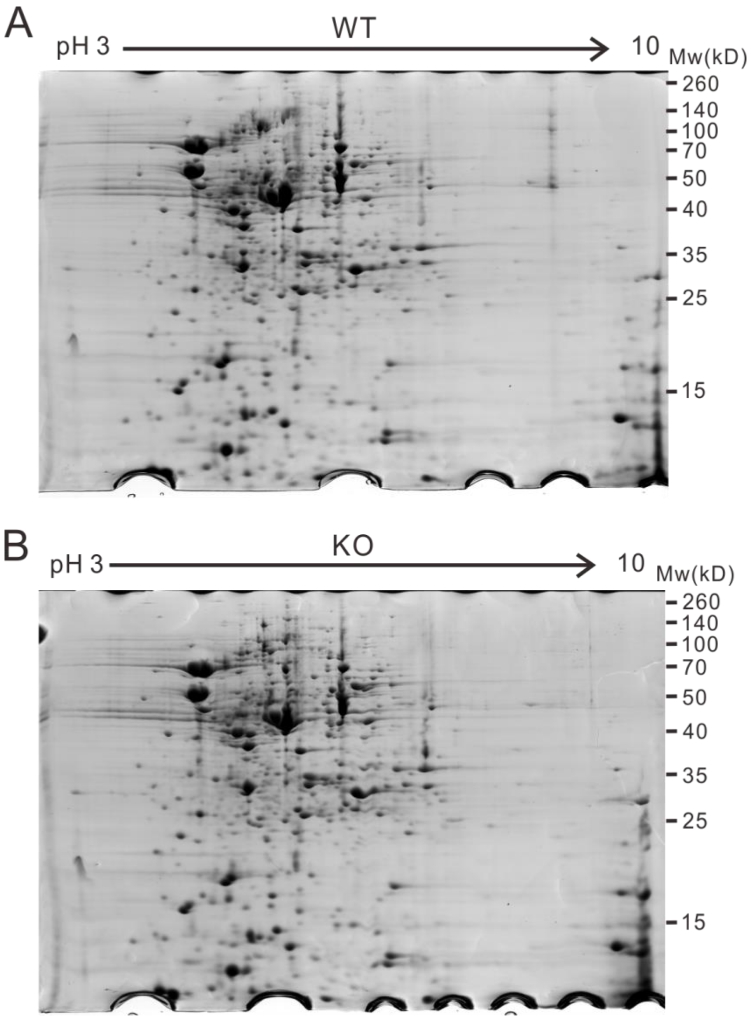

Supplementary Figure S1. 2-DE images of total proteins in *E. coli* at pH 7. Proteins from *E. coli* WT (A) and ΔEF4 (KD) (B) cells were extracted and separated on a pH 3-10 IPG strip, followed by 12% SDS-PAGE and Coomassie Brilliant Blue R-250 staining. There were no detectable differences in spots between the gels.

MATERIALS AND METHODS

All chemicals used were purchased from Sigma-Aldrich unless otherwise indicated.

Bacterial strains and culture conditions

BW25113 (*lacI*^{q} *rrnB*$_{T14}$ Δ*lacZ*$_{WJ16}$ *hsdR514* Δ*araBA-D*$_{AH33}$ Δ*rhaBAD*$_{LD78}$) is a derivative of the F^{-}, λ^{-}, *E. coli* K-12 strain BD792 and was obtained from the Keio collection (National Institute of Genetics, Japan) (Baba et al., 2006). Plasmids pKD46 and pKD13 were kind gifts from Dr. Yong Tao of the Institute of Microbiology, CAS. The ΔEF4 strain was generated as described previously with a few modifications (Baba et al., 2006). The gene was replaced by an in-frame kanamycin resistance gene using the following primer pair: 5'-GCCTGGTTAACCGGGCATTAAGGCACAATAATCATACTTATTCCGGGGATCCGTCGACC-3' and 5'-TCAGGGCAAACATATTCGCCATGCCAACTCCTAAGGGTTTGTAGGCTGGAGCTGCTTCG-3' (underline sequence are homology arms). The kanamycin cassette was produced by PCR amplification from the plasmid pKD13, and the products were transformed into competent cell BW25113 containing pKD46. Recombinants were selected on LB (1% NaCl, 1% tryptone and 0.5% yeast extract) agar-kanamycin (50 μg/ml) plates and further screened by PCR. The kanamycin-resistance gene was deleted by FLP recombinase (Datsenko and Wanner, 2000) which targets the repeated FRT (FLP recognition target) sites flanking the resistance gene.

Complementation of *E. coli* ΔEF4 strains

EF4 rescue plasmid was constructed with plasmid pL11 (tetracycline-resistant) according to previous paper (Van Dyke et al., 2002). The redundant NheI restriction site of pL11 was mutated by PCR amplification using primers 5’-CCTAGCGCTATATGCGTTG-3’ and 5’-AGCACGCCATAGTGACTGG-3’. The *rplK* gene fragment was digestion with restriction enzyme NheI-HF^{TM} (NEB). The *ef4* gene with an additional 20 nt long segment upstream of the 5’ end and 4 nt downstream from the 3’ end was amplified from *E. coli* genomic DNA using primers 5’-TAGCTAGCAGGCACAATAATCATACTTTATGA-3’ and 5’-TAGCTAGCGCCAACTCCTAAGGGTTATT-3’ (underline sequence are NheI sites). The *ef4* gene was cloned into pL11 plasmid without *rplK* gene by T4 DNA ligase. Recombinant plasmid was transformed into *ef4* KO cells and confirmed by sequencing.

Growth of *E. coli* cells under different pH conditions

BW25113 and ΔEF4 cells both transformed a pACYC184 plasmid (tetracycline-resistant) as well as EF4 rescued cells containing the rescued plasmid (tetracycline-resistant) were cultivated on LB agar plates containing 10 μg/ml tetracycline at 37°C overnight. Single colonies were inoculated into 60 ml LB liquid medium and cultivated overnight. Cells were added into 200 ml LB medium under different pH at a dilution of 1:100 with the same OD_{580} and then shaken at 200 rpm (37°C). In addition, IPTG with a final concentration of 0.2 mM was added to LB medium when cells were inoculated. OD_{580} was detected at the indicated intervals. Then Cells at pH 7 and pH 4 were further diluted ×10 with pH 7 medium once the

stationary phase was reached, and OD_{580} was also detected at the indicated intervals.

Preparation and analysis of polysomes

A chloramphenicol-treated polysome fraction was prepared from *E. coli* WT and EF4 KO strains as well as rescued strains at pH 7 and pH 4 as described previously (Ishino et al., 2000) with some modifications. When the OD_{580} reached 0.1, 0.4, 0.6, 0.8 and 1.0 respectively, the cells were treated with $MgCl_2$ (at a final concentration of 6 mM) and chloramphenicol (100 μg/ml) for 2 min, The cultures were immediately placed on ice before collection. Cells were spun down for 10 min at 4°C and 1,600g. The pellet was resuspended in prechilled buffer (20 mM Hepes-KOH pH 7.6, 8.2 mM $MgAc_2$, 80 mM NH_4Ac, 4 mM β-mercaptoethanol). Cells were lysed by freezing and thawing three times. Crude extracts were clarified by centrifugation for 1 h at 4°C and 18,000g. Typically 10 OD_{260} U were loaded on linear 15-45% (w/v) sucrose gradients and spun using SW40 rotor (Beckman) for 3.5 h at 4 ℃ and 160,000g (Karim, 2009). Gradients were manually collected by following the absorption trace at 260 nm, and the quantity of each gradient was calculated.

Assay for protein synthesis by measuring incorporation of ^{35}S-methionine

WT and KO cells at pH 4 and pH 7 were grown in LB medium at 37 ℃. When the OD_{580} reached 0.6, 5 ml of the cultures were transferred to new 50 ml tubes, added with 4 μl EasyTag™ L-Methionine (0.5 mCi/ml) (PerkinElmer) and cultured at 37 ℃. At the time points of 0, 3, 6, 10 min, 1 ml samples were removed and placed on ice containing 100 μl stop solution (100 mM Tris-HCl pH 7.5, 1mg/ml chloramphenicol, 10 mM methionine). All samples were then spun down and washed at least three

times with cold wash buffer (100 mM Tris-HCl pH 7.5, 0.1 mg/mL chloramphenicol, 1 mM methionine). The pellets were lysed in a boiling water bath for 20-30 min, and protein content was determined by BCA kit (Pierce). Then 1 μg protein was used for liquid scintillation counting.

Preparation of protein samples for 2-dimensional electrophoresis (2-DE)

E. coli cells were inoculated into LB medium and cultured overnight at 37 ℃. 5 ml of the cultures was diluted into 250 ml LB medium, pH 4 or pH 7. Cells were grown at 37 ℃ until the OD_{580} reached 0.6. Cells were collected and washed three times with buffer (10 mM Tris, 5 mM $MgAc_2$) before being lysed in lysis buffer (8 M Urea, 4% CHAPS, 65 mM DTT, 1% IPG buffer, 0.5% DNase I). The sample was freeze-thawed once using liquid nitrogen, and then incubated on ice for 30 min. Samples were sonicated for 3 min and centrifuged for 30 min at 4 ℃ and 18,000g. Supernatants were clarified twice by centrifugation for 30 min at 4 ℃ and 23,800g. The protein concentration of each sample was measured on a Model 550 Microplate Reader using an RC DC Protein Assay Kit (Bio-Rad) with BSA as the standard.

SDS-PAGE and 2-DE

1.5 mg protein samples were separated by 12% SDS-PAGE, and then stained with Coomassie Brilliant Blue R-250. 0.7 mg protein samples were separated in the first dimension on a 24 cm, isoelectric focusing (IEF) tray with a pH 3-10 IPG gel strip (GE Healthcare) and by 12% SDS-PAGE in the second dimension. All samples were run at least twice to confirm reproducibility. Analytical gels were stained with Coomassie Brilliant Blue R-250 and then scanned with an UMAX Scanner

(Powerlook 1120). The images were analyzed using ImageJ software.

Mass spectrometry and protein identification

Spots that showed significant differences between the ΔEF4 strain and its mother strain were excised from the gel and digested with trypsin. Peptides were recovered as previously described (Koskenniemi et al., 2009) and then submitted to mass spectrometry. Mass spectra were acquired in positive reflector mode on a Bruker Autoflex III™ MALDI TOF/TOF (Bruker Daltonics 6.0). Uninterpreted mass spectra results were searched against the nonredundant protein sequence database from the National Center for Biotechnology Information (NCBI) using GPS Explorer™ v3.6 software (Applied Biosystems) and the Mascot v2.1 software (Matrix Science). Search parameters were as follows: NCBInr database, *E. coli* retrieval species, one missed trypsin cleavage allowed, non-fixed modifications of methionine (oxidation) and cysteine (carbamidomethylation). Mass error ranges setting: PMF 0.3 Da, MS/MS 0.4 Da. Trypsin self-digestion peaks and impurity contamination peaks in the database search were removed by hand. Proteins quantity was measured by ImageJ software.
